# Teaching, service, and leadership: exploring gender inequities in Saudi pharmacy academia

**DOI:** 10.3389/fmed.2025.1712555

**Published:** 2025-12-11

**Authors:** Dalia Almaghaslah

**Affiliations:** Department of Clinical Pharmacy, College of Pharmacy, King Khalid University, Abha, Saudi Arabia

**Keywords:** gender equity, pharmacy academia, Saudi Arabia, teaching workload, faculty advancement

## Abstract

**Background:**

Gender equity remains a global challenge in academic pharmacy, with disparities documented in rank, leadership, and workload distribution. However, limited evidence exists from Middle Eastern contexts where unique structural and cultural factors, such as gender segregation, shape faculty experiences.

**Objective:**

This study examined gender equity perceptions among faculty at a Saudi college of pharmacy, focusing on workload distribution, access to resources, classroom experiences, and advancement opportunities, and compared findings with national data from the AACP-APhA Gender Equity Task Force.

**Methods:**

A cross-sectional survey adapted from the AACP-APhA Gender Equity Task Force instrument was administered to pharmacy faculty. The survey measured perceptions of equity across domains including teaching, research, service, student interactions, mentoring, leadership, and promotion. Responses were analyzed using chi-square tests to identify gender differences.

**Results:**

Significant gender differences were identified across multiple domains. Female faculty reported heavier teaching workloads, less access to research resources, and greater service obligations compared to male colleagues. They also perceived significant disparities in classroom civility (*p* = 0.0052), student respect, and teaching evaluations (*p* = 0.0451), with students perceived as treating male faculty more favorably. Leadership opportunities, promotion, and peer respect also significantly favored men. These findings are consistent with national data, reinforcing that inequities in academic pharmacy are systemic rather than context-specific.

**Conclusion:**

Women faculty in Saudi pharmacy education face structural and cultural barriers, compounded by implicit student bias, that limit their time for scholarship and affect their career advancement. Institutional interventions, including equitable workload distribution, resource allocation, bias training, and mentorship programs, are essential to promote gender equity and ensure a supportive academic environment.

## Introduction

Pharmacy as a career path has been attracting more women over the last decade. A growing number of female pharmacy students have joined pharmacy colleges around the globe. As a result, more and more women are entering the pharmacy job market across various sectors, including academia (1).

However, despite the growing interest of women in studying and practicing pharmacy, gender disparities have been identified (2).

Despite the growing interest of women in studying and practicing pharmacy, gender disparities have been identified in various pharmacy sectors. Gender equity has been a long-standing issue since women entered the pharmacy job market. Disparities have been reported across many fields, including academia, where unequal compensation, limited opportunities for promotion, and incidents of sexual harassment remain concerns (3, 4).

Other occasions that demonstrate gender inequality include the lower representation of females in academic events compared to their male counterparts. This underrepresentation of female professionals and scientists in such forums naturally limits the visibility of their scholarly achievements within the academic community. Consequently, women face reduced opportunities to compete for and attain leadership positions in academia. In recent years, growing awareness of the gender gap in academic medicine has prompted investigations into the presence of female authors in scientific publications (5, 6)

It is important to understand the concepts of ‘gender equality' and ‘gender equity' when evaluating and working toward their implementation. Gender equality is defined as ‘having the same rights, status, and opportunities as others, regardless of one's gender.' In contrast, gender equity refers to ‘fairness of treatment for women and men, according to their respective needs.' This may involve either equal treatment or different treatment that is deemed equivalent in terms of rights, benefits, obligations, and opportunities (7, 8).

For example, Data from the American Association of Colleges of Pharmacy (AACP) highlighted that female faculty members face significantly reduced chances of advancing to senior academic positions, such as associate professor or dean/CEO roles (9). Additionally, the study reported a meaningful wage disparity, with women earning less per year than their male colleagues (10–13).

From a global perspective, practicing gender equity has been prioritized by the United Nations Sustainable Development Goals (SDGs) as well as the International Pharmaceutical Federation (FIP) Development Goals (14). Although progress has been made to increase women's involvement across different areas, epidemiological data continue to demonstrate that they remain underrepresented in the broader science, technology, and education sectors. At present, gender inequities in the workplace extend beyond mere statistical imbalance between men and women; they represent a substantial challenge at local, national, and global levels (14, 15).

Achieving gender equity requires ensuring that individuals of all genders receive fair rights, responsibilities, treatment, and access to opportunities that reflect their specific needs. Promoting gender equity in the workplace not only enhances organizational performance and public image but also contributes to higher national productivity and stronger economic development (4, 16, 17).

In the Middle East, gender equity is shaped by a range of factors, including historical, cultural, socio-economic, and religious influences, which can further complicate efforts to achieve equality in professional settings such as pharmacy (2).

In Saudi Arabia, women's empowerment is a key initiative launched as part of Saudi Vision 2030 (18, 19). The Ministry of Human Resources and Social Development has actively promoted the participation of women in civil services and leadership roles. This goal is being achieved by increasing women's presence across all government sectors, expanding their professional roles, ensuring equal opportunities for all genders, and advancing women's capabilities and skills through targeted development programs (18, 20).

The aim of the current study is to assess gender equity in academia at a college of pharmacy in Saudi Arabia across the following domains: research, teaching, service, recruitment, mentoring, and advancement

## Methodology

### Study design

This study utilized a cross-sectional design conducted between May 2025 and September 2025.

### Setting

The research was carried out at a College of Pharmacy in Saudi Arabia, targeting faculty members teaching in the Pharm D program at a Saudi government university.

### Participants

The study included pharmacy faculty members involved in teaching undergraduate Pharm D and postgraduate Master's programs at the College of Pharmacy.

### Sample size and sampling procedure

A non-probability convenience sampling method was used to recruit participants. Invitations were initially sent via official university email to all faculty members (*n* = 82). To increase participation, a reminder email was sent 2 weeks after the initial invitation, followed by a second reminder after 1 month. A total of 64 faculty members responded via email. As no further increase in responses was observed after the second reminder, in-person recruitment was conducted to boost participation.

Data collection was conducted through an anonymous online survey that did not request any identifying information such as names or job numbers. The in-person recruitment was only intended to encourage participation and did not involve assigning or recording participant identities.

### Data collection tool

The final instrument consisted of 21 questions assessing perceptions of gender equity across the following academic domains:

- **Research** (3 items: research expectations, grant procurement expectations, and research resources)
- **Teaching** (5 items: teaching workload, classroom civility from students, student respect, teaching evaluations, and teaching resources)
- **Service** (3 items: service workload, service types, and service resources)
- **Recruitment** (3 items: recruitment courtesies, startup package, and salary)
- **Mentoring** (2 items: opportunity to mentor and be mentored)
- **Advancement** (3 items: respect from peers, leadership opportunities, and promotion and tenure)

Additionally, one item assessed the impact of family status on opportunities, and another addressed the influence of the department chair's gender on departmental collegiality. For each question, respondents were asked: “For the following domains in academic life, do you believe there is gender equity?” Responses were recorded on a 5-category scale: *favoring men much more, favoring men more, same for men and women, favoring women more, favoring women much more*, with an additional “I don't know” option. The questionnaire also included demographic questions and queries about personal experiences with gender inequity. The questionnaire is available upon request from the corresponding author.

The 21-item questionnaire used in this study was developed and validated by Planas et al. (16) to explore perceptions of gender equity among pharmacy staff members in academia.

### Data analysis

All data were coded and analyzed using SPSS version 26.0 for Mac. Descriptive statistics, including frequencies and percentages, were used to summarize the data. Chi-square (χ^2^) tests of independence (α = 0.05) assessed the relationship between respondents' gender and both gender inequity experiences and perceptions of gender equity. For gender equity perceptions measured on a 5-category scale, *post hoc z*-tests with Bonferroni-adjusted *p*-values were used to identify specific response categories showing significant gender differences.

### Ethics approval

Ethical approval was granted by the Ethics Committee at King Khalid University (ECM# 2025-402).

## Results

Participant characteristics are summarized in Table 1. Just over half of the participants were male (34, 53.1%). The majority held a PhD as their terminal degree (56, 87.5%). Approximately three-quarters of respondents (45, 70.3%) had obtained their most recent degree within the past 1–10 years. More than half of the faculty members (36, 56.2%) were assistant professors, while nearly one-third (20, 31.2%) were associate professors. The mean age of participants was 39.8 years (SD = 5.8).

**Table 1:** Participant demographic and academic characteristics.

**Variable**	**Category**	**Frequency (%)**
Gender	Male	34 (53.1%)
	Female	30 (46.9%)
Terminal Degree	PhD	56 (87.5%)
	PharmD	8 (12.5%)
Years Since Degree	1–10	45 (70.3%)
	11–20	16 (25.0%)
	21–30	2 (3.1%)
	>30	1 (1.6%)
Academic Rank	Assistant professor	36 (56.2%)
	Associate professor	20 (31.2%)
	Professor	3 (4.7%)
	Teaching assistant	3 (4.7%)
	Lecturer	2 (3.1%)
Age	Mean ± SD	39.8 ± 5.8

Of the 64 participants, 14 (21.9%) were reported experiencing gender inequity based on the majority rule cutoff. Experiencing gender inequity was significantly associated with gender, with 36.7% of women reporting inequity compared with 8.8% of men (p = 0.017). No significant associations were found between experiences of gender inequity and years since degree, academic rank, or terminal degree (Table 2).

**Table 2:** Gender inequity experiences.

**Variable**	**Category**	**No *n*(%)**	**Yes *n*(%)**	**Total**	**p-value ^a^**
Gender	Female	19 (63.3%)	11 (36.7%)	30	0.017
	Male	31 (91.2%)	3 (8.8%)	34	
Years since degree	1-10	33 (73.3%)	12 (26.7%)	45	0.5188
	11-20	14 (87.5%)	2 (12.5%)	16	
	21-30	2 (100.0%)	0 (0.0%)	2	
	>30	1 (100.0%)	0 (0.0%)	1	
Academic rank	Assistant professor	30 (83.3%)	6 (16.7%)	36	0.2011
	Associate professor	15 (75.0%)	5 (25.0%)	20	
	Lecturer	1 (50.0%)	1 (50.0%)	2	
	Professor	1 (33.3%)	2 (66.7%)	3	
	Teaching assistant	3 (100.0%)	0 (0.0%)	3	
Terminal degree	PhD	42 (75.0%)	14 (25.0%)	56	0.2531
	Pharm D	8 (100.0%)	0 (0.0%)	8	

^a^χ2 test of independence (α = 0.05); Fisher exact test (α = 0.05) if cell size < 5.

Frequencies and percentages of aggregate responses to gender equity perception items are presented in Table 3. Overall, the most frequently selected response was that women and men are treated equally, which was observed for the majority of items. However, gender was significantly associated with responses to 13 of the 21 items.

**Table 3:** Research, Teaching, Service, Recruitment, Mentoring, and Advancement Gender Equity Perceptions (*N* = 64).

**Item**	**Gender**	**Favoring men much more**	**Favoring men more**	**Same for men and women**	**Favoring women more**	**Favoring women much more**	***p*-value**
Research expectations,	Male	0 (0.0%)	0 (0.0%)	32 (97.0%)	0 (0.0%)	1 (3.0%)	0.0002
	Female	4 (14.8%)	6 (22.2%)	14 (51.9%)	3 (11.1%)	0 (0.0%)	
Grant procurement expectations,	Male	0 (0.0%)	1 (3.1%)	29 (90.6%)	2 (6.2%)	0 (0.0%)	0.2
	Female	1 (4.3%)	2 (8.7%)	20 (87.0%)	0 (0.0%)	0 (0.0%)	
Resources for research	Male	0 (0.0%)	4 (12.1%)	28 (84.8%)	0 (0.0%)	1 (3.0%)	0.007
	Female	5 (20.0%)	6 (24.0%)	13 (52.0%)	1 (4.0%)	0 (0.0%)	
Teaching workload	Male	0 (0.0%)	0 (0.0%)	30 (90.9%)	1 (3.0%)	2 (6.1%)	0.003
	Female	4 (15.4%)	7 (26.9%)	14 (53.8%)	0 (0.0%)	1 (3.8%)	
Classroom civility from students	Male	0 (0.0%)	0 (0.0%)	28 (87.5%)	4 (12.5%)	0 (0.0%)	0.003
	Female	5 (20.0%)	3 (12.0%)	12 (48.0%)	4 (16.0%)	1 (4.0%)	
Student respect	Male	1 (2.9%)	1 (2.9%)	29 (85.3%)	2 (5.9%)	1 (2.9%)	0.02
	Female	2 (7.7%)	6 (23.1%)	15 (57.7%)	3 (11.5%)	0 (0.0%)	
Teaching evaluation	Male	1 (3.0%)	0 (0.0%)	29 (87.9%)	3 (9.1%)	0 (0.0%)	0.2
	Female	1 (4.3%)	4 (17.4%)	18 (78.3%)	0 (0.0%)	0 (0.0%)	
Resources for teaching	Male	0 (0.0%)	3 (8.8%)	28 (82.4%)	1 (2.9%)	2 (5.9%)	0.2
	Female	4 (15.4%)	3 (11.5%)	19 (73.1%)	0 (0.0%)	0 (0.0%)	
Service workload	Male	1 (3.0%)	5 (15.2%)	23 (69.7%)	3 (9.1%)	1 (3.0%)	0.2
	Female	2 (8.3%)	3 (12.5%)	18 (75.0%)	1 (4.2%)	0 (0.0%)	
Service types	Male	0 (0.0%)	5 (16.1%)	25 (80.6%)	1 (3.2%)	0 (0.0%)	0.03
	Female	6 (24.0%)	3 (12.0%)	15 (60.0%)	1 (4.0%)	0 (0.0%)	
Resources for service	Male	0 (0.0%)	5 (15.6%)	26 (81.2%)	1 (3.1%)	0 (0.0%)	0.06
	Female	5 (20.0%)	5 (20.0%)	14 (56.0%)	1 (4.0%)	0 (0.0%)	
Courting during recruitment	Male	1 (3.6%)	1 (3.6%)	24 (85.7%)	1 (3.6%)	1 (3.6%)	0.007
	Female	8 (36.4%)	4 (18.2%)	9 (40.9%)	1 (4.5%)	0 (0.0%)	
Startup package	Male	1 (2.9%)	4 (11.8%)	26 (76.5%)	2 (5.9%)	1 (2.9%)	0.007
	Female	4 (18.2%)	3 (13.6%)	15 (68.2%)	0 (0.0%)	0 (0.0%)	
Salary	Male	1 (3.1%)	2 (6.2%)	26 (81.2%)	1 (3.1%)	2 (6.2%)	0.5
	Female	2 (8.0%)	6 (24.0%)	17 (68.0%)	0 (0.0%)	0 (0.0%)	
Opportunity to mentor	Male	1 (2.9%)	6 (17.6%)	26 (76.5%)	1 (2.9%)	0 (0.0%)	0.01
	Female	9 (32.1%)	3 (10.7%)	16 (57.1%)	0 (0.0%)	0 (0.0%)	
Opportunity to be mentored	Male	2 (6.1%)	2 (6.1%)	28 (84.8%)	1 (3.0%)	0 (0.0%)	0.006
	Female	3 (10.7%)	6 (21.4%)	13 (46.4%)	6 (21.4%)	0 (0.0%)	
Respect from peers	Male	1 (2.9%)	1 (2.9%)	30 (88.2%)	2 (5.9%)	0 (0.0%)	0.04
	Female	7 (25.0%)	3 (10.7%)	18 (64.3%)	0 (0.0%)	0 (0.0%)	
Leadership opportunities	Male	2 (6.1%)	10 (30.3%)	20 (60.6%)	1 (3.0%)	0 (0.0%)	0.008
	Female	11 (39.3%)	11 (39.3%)	6 (21.4%)	0 (0.0%)	0 (0.0%)	
Promotion and tenure	Male	1 (2.9%)	1 (2.9%)	31 (91.2%)	1 (2.9%)	0 (0.0%)	0.001
	Female	6 (22.2%)	6 (22.2%)	15 (55.6%)	0 (0.0%)	0 (0.0%)	
How family status affects opportunities	Male	2 (6.5%)	8 (25.8%)	18 (58.1%)	2 (6.5%)	1 (3.2%)	0.2
	Female	6 (25.0%)	7 (29.2%)	8 (33.3%)	1 (4.2%)	2 (8.3%)	
The impact of the department chair's gender on department collegiality	Male	2 (6.2%)	7 (21.9%)	23 (71.9%)	0 (0.0%)	0 (0.0%)	0.3
	Female	5 (20.8%)	5 (20.8%)	13 (54.2%)	0 (0.0%)	1 (4.2%)	

For research expectations, 97.0% of male respondents reported equal treatment compared with 51.9% of female respondents (*p* = 0.0004). Similarly, 84.8% of men vs. 52.0% of women perceived equal resources for research (*p* = 0.0173). Teaching workload (90.9% men vs. 53.8% women, *p* = 0.0015) and classroom civility from students (87.5% men vs. 48.0% women, *p* = 0.0052) also showed large gender differences. For teaching evaluations, 87.9% of men compared with 78.3% of women perceived equality (*p* = 0.0451).

In service-related domains, male respondents more frequently indicated equality than females for service types (80.6% vs. 60.0%, *p* = 0.0375) and resources for service (81.2% vs. 56.0%, p = 0.049). Recruitment processes also differed significantly, with 85.7% of men vs. 40.9% of women reporting equal treatment (*p* = 0.0057).

For mentoring, male respondents more often perceived equality in opportunities to mentor (76.5% men vs. 57.1% women, *p* = 0.0162) and to be mentored (84.8% men vs. 46.4% women, *p* = 0.0122). Similar gender differences were observed for respect from peers (88.2% men vs. 64.3% women, *p* = 0.0185), leadership opportunities (60.6% men vs. 21.4% women, *p* = 0.0023), and promotion and tenure (91.2% men vs. 55.6% women, *p* = 0.0045).

In each of these areas, male respondents were more likely to indicate that women and men are treated equally, whereas female respondents more often perceived advantages for men. No significant gender differences were noted for grant procurement expectations, resources for teaching, service workload, startup package, salary, family status affecting opportunities, or the impact of the department chair's gender on collegiality

## Discussion

This study examined experiences and perceptions of gender equity among pharmacy faculty. Of the 64 participants, 21.9% were classified as having experienced gender inequity, with significantly more women than men reporting inequity. These findings align with previous literature that has documented disproportionate challenges for women in academic pharmacy (1, 21).

Perceptions of gender equity varied substantially across academic domains. Gender was significantly associated with responses to 13 of the 21 survey items. In particular, women more frequently reported inequities in research expectations, access to resources, teaching workload, classroom civility, and advancement opportunities, while men were more likely to perceive equal treatment across these areas. These results mirror earlier observations that men often underrecognize gender inequities that women experience. A consistent theme in our data, as in prior studies, was that family status is perceived to disadvantage women more than men, although men and women differed in whether this was perceived as a “slight” or “much greater” disadvantage.

In terms salaries, participants of all genders reported no differences between men and women. Since there is a unified salary scale for faculty members in Saudi universities, no differences in salaries exist between male and female faculty. This result contrasts with findings from the American Association of Colleges of Pharmacy (AACP), which continue to show significant gender disparities in faculty salary (7, 11, 22). Startup packages may differ, particularly for international faculty. These variations are sometimes used as a strategy to attract uniquely qualified or highly sought-after candidates.

A significant finding from our study is that women faculty are more likely to be engaged in routine tasks, such as teaching, and often carry heavier teaching workloads while having less access to instructional and research resources. This imbalance may be partly explained by the gender-segregated structure of the institution, where male and female students are taught on separate campuses. With the number of female students being considerably higher, female faculty consequently take on a larger share of teaching responsibilities, although some courses are delivered by male faculty to female students. This result is consistent with previous literature, which has noted that female faculty often face imbalances in the distribution of research, teaching, and service responsibilities (23). Women in academic pharmacy, in particular, tend to devote a larger share of their time to service activities compared to men, leaving them with disproportionately less time for scholarship. It remains unclear whether this is due to women being formally assigned more service responsibilities or whether cultural and institutional pressures encourage women to volunteer for these roles. Regardless of the underlying cause, having less time than their male colleagues to engage in research activities places women at a disadvantage in the promotion and tenure process.

Leadership patterns in our study reflected those observed in Western academic pharmacy culture, where significant gender disparities in leadership positions persist (3, 9, 11, 24). Participants reported that leadership roles tended to favor men within the hierarchical structure. At the institution where this study was conducted, all previous deans and current department heads have been male, while female faculty primarily serve as associate department chairs. However, an important question remains: does this reflect women's personal preference, or is it the result of cultural norms and historical hierarchical regulations that have limited women's participation in leadership roles?

Another explanation is the limited presence of female role models. In pharmacy academia, women remain underrepresented in senior leadership, and as a result, the academic community is less familiar with women occupying positions of influence and authority (3, 25). Our findings reflected this gap, as participants noted insufficient opportunities for guidance and mentorship. The shortage of women in leadership reduces access to advocates who can support professional development and career advancement. Furthermore, balancing professional and personal responsibilities continues to be a significant obstacle, especially for young mothers and women who often assume primary caregiving duties, thereby placing their careers in a secondary role.

In our study, although the findings varied regarding how family status influences career opportunities, more women reported being affected by family obligations compared to their male counterparts. Similar to previous reports, women in academic pharmacy face greater challenges in achieving work–life balance and express higher intentions to leave the profession (26, 27).

Taken together, these findings suggest that structural inequities persist in pharmacy academia, particularly in areas related to research resources, teaching and service burden, mentoring, leadership, and promotion. Raising awareness among faculty, especially male faculty, may be essential for fostering inclusive academic environments. Colleges and schools of pharmacy should consider climate surveys, equity training, and intentional mentoring programs to address identified disparities and ensure equitable advancement pathways (28).

In the context of Saudi Arabia, these findings align with national reform efforts under the Saudi Vision 2030, which has actively encouraged women's participation in the labor market. This national initiative aims to increase women's representation across all government sectors and job levels by investing in their skills and capabilities, expanding employment opportunities, ensuring equal opportunities for all genders, and empowering women to assume senior leadership positions in government institutions through a range of supportive programs.

Our findings revealed statistically significant gender differences in both classroom civility and student respect. Female faculty were far more likely than male faculty to report that students behaved more civilly and respectfully toward male instructors, while most male faculty perceived treatment as equal. These results mirror national findings from the AACP-APhA Task Force, which also documented that women perceive male faculty as receiving more favorable treatment in classroom interactions, whereas men largely fail to recognize such disparities (16). Collectively, these findings underscore the role of implicit student bias in shaping faculty experiences, with potential consequences for women's teaching evaluations, professional recognition, and advancement opportunities.

Overall, the findings of this study closely align with the national AACP–APhA Taskforce report as represented in the study by Planas et al. (16), with women in both settings reporting disadvantages in research support, teaching workload, classroom civility, mentoring, leadership opportunities, and promotion, while men were more likely to perceive equal treatment. In our sample, 36.7% of women reported experiencing inequity compared with 8.8% of men, whereas the U.S. study reported much higher rates (90.9% of women and 37.5% of men). Unlike the U.S. context, where notable salary disparities were documented, no salary inequity was observed in the Saudi setting due to the unified national pay scale. Additionally, structural factors such as gender-segregated campuses and larger female student cohorts contributed to heavier teaching burdens for women. Despite contextual differences, both studies highlight persistent systemic barriers that limit women's advancement in academic pharmacy.

Limitations of this study include the modest sample size and potential response bias, as participation may have been higher among faculty interested in or directly affected by issues of gender equity. Nonetheless, this study contributes valuable insight into the lived experiences and perceptions of gender equity among pharmacy faculty and provides an important foundation for institutional strategies aimed at addressing inequities.

## Conclusion

This study highlights persistent gender inequities within a Saudi college of pharmacy, reflecting patterns also documented internationally. Women faculty reported heavier teaching workloads, limited access to research resources, and a greater share of service responsibilities, leaving them with less time for scholarly activities essential for promotion and tenure. They also perceived significant disparities in classroom civility, student respect, and teaching evaluations, with students more likely to treat male faculty favorably. These perceptions align with national findings from the AACP-APhA Task Force, suggesting that inequities in academic pharmacy are systemic rather than context-specific. Institutional programs that align with Saudi Vision 2030 in promoting women's empowerment should be considered to achieve the vision's goals.

## Data Availability

The raw data supporting the conclusions of this article will be made available by the authors, without undue reservation.
