# Supplementary material for: Teaching, service, and leadership: exploring gender inequities in Saudi pharmacy academia
Source: Front Med (Lausanne). 2025 Dec 11;12:1712555. doi: 10.3389/fmed.2025.1712555 (PMC12738321; doi:10.3389/fmed.2025.1712555)
Supplement: Supplementary file 1 [file Table_1.docx]

Please answer the following question

Demographics and background information

1. Gender
2. Male
3. Female
4. Age …..
5. Terminal degree
6. Pharm D
7. PhD
8. Other
9. Years since terminal degree
10. 1-10
11. 11-20
12. 21-30
13. >30
14. Academic rank
15. Teaching assistant
16. Lecturer
17. Assistant professor
18. Associate professor
19. Professor

Rate the following statement addressing gender equity perceptions in the following academic domains

1. Research

|  | favoring men much more | favoring men more | same for men and women | favoring women more | favoring women much more. | I don’t know |
| --- | --- | --- | --- | --- | --- | --- |
| Research expectations |  |  |  |  |  |  |
| Grant procurement expectations |  |  |  |  |  |  |
| Resources for research |  |  |  |  |  |  |

1. Teaching

|  | favoring men much more | favoring men more | same for men and women | favoring women more | favoring women much more. | I don’t know |
| --- | --- | --- | --- | --- | --- | --- |
| teaching workload |  |  |  |  |  |  |
| classroom civility from students |  |  |  |  |  |  |
| student respect |  |  |  |  |  |  |
| teaching evaluations, |  |  |  |  |  |  |
| resources for teaching |  |  |  |  |  |  |

1. Service

|  | favoring men much more | favoring men more | same for men and women | favoring women more | favoring women much more. | I don’t know |  |
| --- | --- | --- | --- | --- | --- | --- | --- |
| service workload |  |  |  |  |  |  |  |
| service types |  |  |  |  |  |  |  |
| resources for service |  |  |  |  |  |  |  |

1. Recruitment

|  | favoring men much more | favoring men more | same for men and women | favoring women more | favoring women much more. | I don’t know |
| --- | --- | --- | --- | --- | --- | --- |
| courting during recruitment |  |  |  |  |  |  |
| startup package |  |  |  |  |  |  |
| salary |  |  |  |  |  |  |

1. Mentoring

|  | favoring men much more | favoring men more | same for men and women | favoring women more | favoring women much more. | I don’t know |
| --- | --- | --- | --- | --- | --- | --- |
| opportunity to mentor |  |  |  |  |  |  |
| opportunity to be mentored |  |  |  |  |  |  |

1. Advancement

|  | favoring men much more | favoring men more | same for men and women | favoring women more | favoring women much more. | I don’t know |
| --- | --- | --- | --- | --- | --- | --- |
| respect from peers |  |  |  |  |  |  |
| leadership opportunities |  |  |  |  |  |  |
| promotion and tenure |  |  |  |  |  |  |

1. Other

|  | favoring men much more | favoring men more | same for men and women | favoring women more | favoring women much more. | I don’t know |
| --- | --- | --- | --- | --- | --- | --- |
| how family status affects opportunities |  |  |  |  |  |  |
| the impact of the department chair’s gender on department collegiality. |  |  |  |  |  |  |
